# Randomized placebo controlled trial of phytoterpenes in DMSO for the treatment of plantar fasciitis

**DOI:** 10.1038/s41598-024-65979-1

**Published:** 2024-07-31

**Authors:** Briant E. Burke, Jon E. Baillie

**Affiliations:** Institute for Biomedical Sciences, 967 East Parkcenter Blvd, Ste 205, Boise, ID 83706 USA

**Keywords:** Diseases, Medical research

## Abstract

Plantar fasciitis is the most common cause of heel pain in adults with an overall prevalence of 0.85% in the adult population of the US, affecting over 2 million adults annually. Most current treatment modalities are not supported by sufficient evidence to recommend one particular strategy over another. Topical application of analgesics for soft tissue pain is well established, however the plantar fascia presents challenges in this regard due to thick skin, fibrotic tissue, and an often thickened fat pad. Sixty-two patients with plantar fasciitis were randomized to a placebo controlled trial testing the efficacy of a topical solution of plant terpenes containing camphor, menthol, eugenol, eucalyptol, and vanillin. Skin permeation of the mixture was enhanced with 15% dimethylsulfoxide (DMSO), 1% limonene, and rosemary oil. One ml of solution was applied topically twice daily, and pain scores evaluated on Day 0, Day 1, Day 3, and Day 10. Using the validated foot function index 78.1% of patients reported an 85% or greater decrease in their total pain score by day 10 while placebo treatment was without effect (One Way ANOVA, P < 0.01). This study adapts the treatment modality of topical analgesia for soft tissue pain to a problematic area of the body and shows therapeutic promise.

ClinicalTrials.gov Identifier: NCT05467631

## Introduction

Plantar fasciitis (PF) is an overuse syndrome resulting in degenerative changes of the fascia at its attachment to the calcaneus, where it originates. It is the most common cause of heel pain in adults^1,2^ with an overall prevalence of 0.85%^3^. Ethnic variation is minimal according. Hispanic whites have the highest prevalence of plantar fasciitis at 0.95%, followed by non-Hispanic whites at 0.93%, and 1.1% in black populations^3^. The lifetime incidence is estimated at 10%^4^. Risk factors for PF include^5,6^ limited ankle dorsiflexion which causes the foot to over pronate and stress the fascia, BMI greater than 27 kg/m^2^, occupations requiring prolonged standing or walking such as the military, sedentary lifestyle, and excessive running where it occurs with an incidence of 5–10%^5^. Plantar fasciitis is a clinical diagnosis made by patient history and physical exam. The classic presentation is sharp stabbing pain in the anteromedial aspect of the heel when first stepping out of bed in the morning^7^. Pain is also present with weight bearing after a period of rest and improves somewhat as walking continues. Paresthesia is uncommon and its presence should raise suspicion of possible Baxter’s nerve entrapment (entrapment of the first branch of the lateral plantar nerve) rather than PF^8^.

Over 75% of patients with PF will improve with conservative non-operative treatment within 12 months^9^. This generally consists of some combination of decreased weight bearing, ice massage, NSAIDs, stretching, strengthening regimen, and heel padding^1^. However, most treatment modalities are not supported by sufficient evidence to recommend one particular strategy over another^1,10^, highlighting the need for a more clearly defined and rapid treatment.

The impact of PF on patient quality of life and lost work should not be underestimated^11^. In the National Health and Wellness Survey of 75,000 participants, of those reporting pain from PF (0.85%), more than 61 percent reported having pain every day, and almost 54 percent reported that their pain interfered with normal work activities at least moderately. Nearly one-third reported severe (“quite a bit” or “extreme”) pain-related interference^3^.

The use of topical analgesics for soft tissue pain is well established, however it has not found widespread use in the treatment of PF. We investigated the application of a topical solution of the plant terpenes camphor, menthol, eugenol, eucalyptol, and vanillin, formulated for enhanced skin permeation, adapting the established use of topical analgesics for soft tissue pain control to a body area with unique challenges. These terpene agents have analgesic, anti-nociceptive, and anti- inflammatory activity^12^. The cohort consisted of 62 patients with PF in a placebo controlled blinded trial. Using the pain subsection of the validated foot function index^13^ as modified^14^, 78.1% of patients had an 85% or greater decrease in their subjective pain score after 10 days of twice daily application.

## Methods

**Figure 1:**
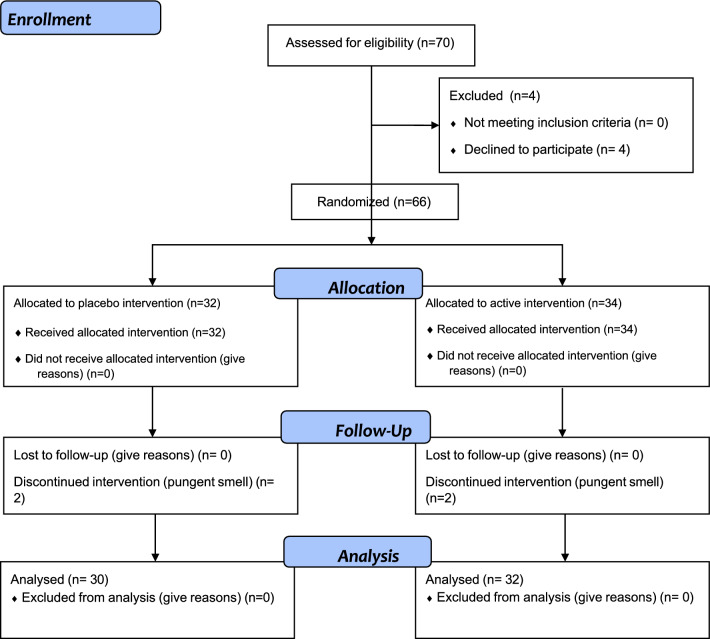
Enrollment algorithm for 10-day endpoint.

This trial was conducted in compliance with institutional guidelines for clinical trials and in accordance with the principles of the Declaration of Helsinki, 2013 revision, on research with human subjects. This study was approved by the Institute for Biomedical Sciences institutional review board and registered 10/26/2021 (ClinicalTrials.gov Identifier: NCT05467631). Detailed informed consent was obtained from each patient and archived. Figure 1 shows the CONSORT flowchart.

Patients 18 years of age or older with physician-diagnosed plantar fasciitis were recruited from a primary care practice and a physical therapy clinic between 01/15/2019 through 4/21/2020 and randomized into a placebo controlled blinded trial on a rolling basis. Patients who had undergone surgery, steroid injection, or low level laser therapy for their PF were excluded from the study, as were patients with diabetes, peripheral neuropathy, RA, or history of foot trauma in the past 6 months. Patients from the physical therapy clinic were recruited during their first visit and prior to initiation of any clinician supervised stretching regimen. Data on NSAID use in the prior month, BMI, OTC shoe insert use, and home stretching program were collected.

The active formulation (US Patent # 10,980,856) contained a mixture of essential oils so that the final formulation was standardized to 10% camphor from rosemary oil, 5% menthol from peppermint oil, 5% eugenol from clove oil, 2% eucalyptol (1,8-cineole) from eucalyptus oil, and 3% vanillin in a base of 10% (v/v) tea tree oil (M. alternifolia), with 15% DMSO, 1% limonene, and rosemary oil as skin permeation enhancers. Placebo consisted of 10% (v/v) tea tree oil in canola oil as a carrier. All materials were obtained from Sigma Chemical Co, St. Louis, MO and were USP grade.

Sixty-two patients, 27 male and 35 female, completed the protocol, and study results for this cohort of patients are presented. Comparison of group means at each time point was performed by one way ANOVA with post-hoc Tukey means testing. Sample size was determined using means and sample calculation software (www.samplesize.net). Patients were randomized by blindly choosing a token numbered 1-10 out of a box. Thirty-two patients chose odd numbered tokens and were randomized to the placebo group and 34 who chose even numbered tokens to the active treatment group. Two patients in each treatment arm withdrew citing the pungent aroma of the formulation as the reason. Patients were blinded to allocation group. The placebo and active formulations did not have identical aromas; however it is unlikely that this had any impact on the blinding of the study protocol. Tea tree oil was the main constituent of each, so the smell was very similar. The fact that each formulation contained essential oils and had an aroma gave no information to any participant about which treatment group they were allocated to. In addition, no patient had exposure to both formulas and would therefore not have any basis for comparison. Follow up on Day 1, 3, and 10 was by phone with the office caller blinded to patient status. Patients were seen in person on Day 14, after the trial had concluded.

Upon enrollment, patients rated their pain over the previous 10 days using the validated Foot Function Index, FFI^13^, modified to a 5point verbal rating scale^14^. All patients completed the FFI-5pt upon enrollment (Day 0). The items of the FFI-5pt are rated on a 5point verbal rating scale, “no pain” (0) to “intense pain” (4) on the pain scale. The FFI-5pt is a suitable generic self-administered measurement of the effect of foot complaints on foot function, with good test-retest reliability^14^. The item scores are summed, divided by the maximum possible score, which is 20, then multiplied by 100. This normalizes the scores to a percentage of the maximum possible pain score. Patients were asked to complete the FFI-5pt on Day 0, Day 1, Day 3, and Day 10. Participants rated their pain on five questions with the largest score for each question being 4 and the maximum total score 20.

1. Foot pain at worst
2. Foot pain in the morning
3. Pain walking barefoot
4. Pain standing barefoot
5. Foot pain end of the day

**Figure 2:**
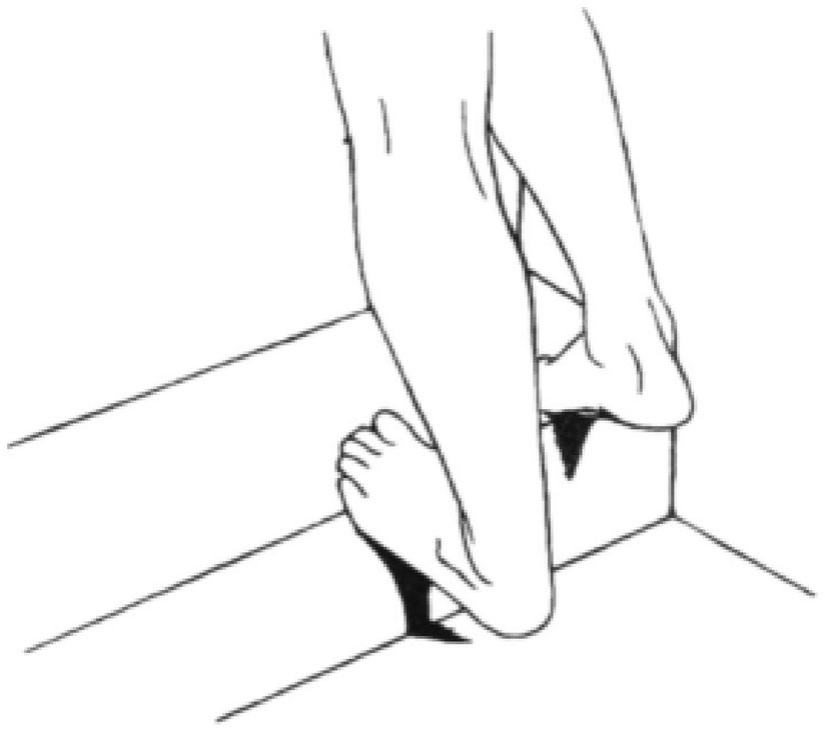
Calf and Achilles Tendon Stretching Exercise.

Patients were instructed to wash the bottom of the affected foot prior to application with soap and water, pat dry, then apply 1ml of placebo or active treatment solution to the bottom of the heel of the affected foot using a calibrated dropper, then spread the solution with a small cuticle brush attached to the bottle cap. Application was twice a day. Patients were seen in follow up 1 day (2 applications), 3 days (6 applications) and 10 days (20 applications) from the date of enrollment (Day 0). All subjects were instructed in a calf/Achilles tendon stretching exercise to be performed twice a day (Fig. 2). Analysis was for participants completing the study. The 4 patients who withdrew were not included in the analysis.

Each patient was instructed to gently stretch each foot, hold for 5 s, then stretch the other foot for a total of 10 stretches each side, twice a day.

## Statistics

Using sample size calculator at https://sample-size.net/means-effect-size/

Using 30 subjects per study arm the study has 80% power to detect an effect size of 0.368.

Threshold probability for rejecting the null hypothesis. Type I error rate: α (two-tailed) = 0.05.

Probability of failing to reject the null hypothesis under the alternative hypothesis. Type II error rate: β = 0.2

Standard deviation of the outcome in the population: S = 0.5

Number of subjects in Group 1: N_1_ = 30.

Number of subjects in Group 0: N_0_ = 30

$${\text{Total group size }} = {\text{ N}}_{{{\text{total}}}} = {\text{ N}}_{{1}} + {\text{ N}}_{0} = {6}0$$

$${\text{Proportion of subjects in Group 1 }} = {\text{ q}}_{{1}} = {\text{ N}}_{{1}} /{\text{ N}}_{{{\text{total}}}} = 0.{5}00$$

$${\text{Proportion of subjects in Group }}0 \, = {\text{ q}}_{0} = { 1 } - {\text{ q}}_{{1}} = 0.{5}00$$

## Calculation using the T statistic and non-centrality parameter:

Degrees of freedom = DoF = N_total_—2 = 58.

The standard T value (with DoF degrees of freedom) corresponding to α = T_α_ = 2.002

$${\text{k }} = \, \surd {1}/{\text{N}}_{{1}} + { 1}/{\text{N}}_{0} = 0.{2582}$$

Non-centrality parameter = δ = 2.8491

$${\text{E}}/{\text{S }} = {\text{ k }}* \, \delta \, = 0.{7356}$$

Data for 62 subjects was available for analysis.

There were 8 group means to be compared one to another: Placebo at Day 0, 1, 3, 10 and Active at Day 0,1,3,10.

One way ANOVA was performed testing group means for statistical significance using the software online at https://statpages.info/anova1sm.html. Confidence level for post-hoc confidence intervals was 95%.

**Table:** 

	Placebo	Active
Mean Pain Score – Day 0	(Grp 1) 65.4 ± 11.6	(Grp5) 61.1 ± 13.6
Mean Pain Score—Day 1	(Grp2) 48.3 ± 14.8	(Grp6) 36.0 ± 10.4
Mean Pain Score—Day 3	(Grp3) 51.9 ± 12.8	(Grp7) 26.8 ± 7.6
Mean Pain Score – Day 10	(Grp4) 60.8 ± 9.2	(Grp8) 8.8 ± 5.2

## Results

Group means testing results are shown in Table 1 and Table 2. Group characteristics are shown in Table 3. The mean age for the active treatment group was 46 ± 12.9 yrs. and for the placebo group 49 ± 10.4 yrs. There was no statistical age difference between the groups. Average duration of symptoms was 4 ± 4.4 months in the placebo group and 5 ± 2.7 in the active group and was not statistically different between the two groups (Table 3). Symptom duration was consistent with the cohort representing patients predominantly in a chronic condition. Participants with pain less than 3 weeks duration were considered acute and accounted for 14.5% (9/62) of the cohort, too small a number to permit comparison of results by pain duration.

**Table 1:** ANOVA Table.

Source Variation	Sum of Squares	d.f	Variance	F	p
Between Groups	85089.8568	7	12155.6938	3.2462	0.0026
Within Groups	898706.2400	240	3744.6093		
Total	983796.0968	247

**Table 2:** Tukey Post-hoc Results p Values.

	Grp 1	Grp2	Grp3	Grp4	Grp5	Grp6	Grp7	Grp8
Grp1	–	p = 0.9	p = 0.9	p = 1.0	p = 1.0	p = 0.6	p = 0.2	p = 0.008
Grp2	–	–	p = 1.0	p = 1.0	p = 0.9	p = 0.9	p = 0.9	p = 0.2
Grp3	–	–	–	p = 1.0	p = 1.0	p = 0.9	p = 0.7	p = 0.11
Grp4	–	–	–	–	p = 1.0	p = 0.8	p = 0.4	p = 0.02
Grp5	–	–	–	–	–	p = 0.7	p = 0.3	p = 0.016
Grp6	–	–	–	–	–	–	p = 1.0	p = 0.6
Grp7	–	–	–	–	–	–	–	p = 0.9

**Table 3:** Study Participant Characteristics.

	Placebo	Active
Mean Age (yrs.)	49 ± 10.4	46 + 12.9
Ave Pain Duration (mo.)	4 ± 4.4	5 ± 2.7
BMI	29.3 ± 1.6	28.6 ± 1.3
Gender	14 M/16F	13 M/19F
Any OTC NSAID use past month	63.3% (19/30)	56.2% (18/32)
OTC insert use past 3mo	100%	100%
Home stretching/massage past mo	73.3% (22/30)	68.8% (22/32)

The mean score upon enrollment for the placebo group was 65.4 ± 11.6 and 61.1 ± 13.6 for the active group. There was no difference in mean pain scores between the two groups at the start of the study. After 10 days of treatment with placebo, the mean pain score was 60.8 ± 9.2, and was not statistically different from mean scores prior to placebo treatment. The mean pain score for the active treatment group after 10 days of treatment decreased to 8.8 ± 5.2. This was statistically significant at p<0.01 compared to placebo treatment (One Way ANOVA; post-hoc Tukey p<0.01). There were no differences in mean pain scores between the placebo before, placebo after, and active before groups.

There was a noticeable decrease in the mean pain scores in the active group on Days 1 and 3, but the differences in pain scores did not reach statistical significance until measured on Day

**Figure 3:**
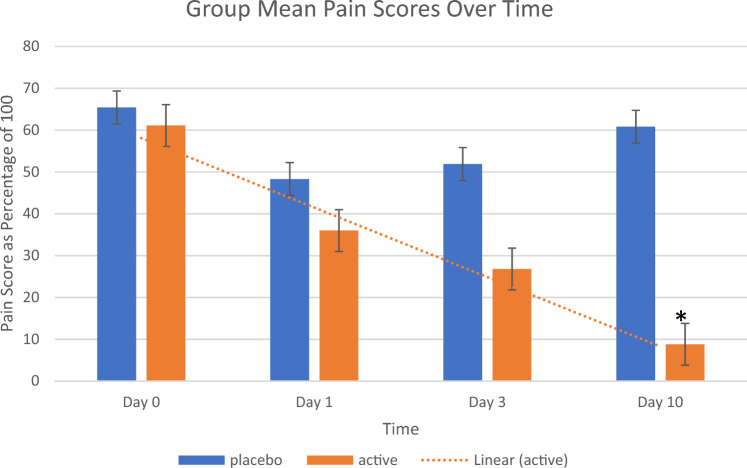
Pain Scores Over Time. Pain scores of 62 plantar fasciitis patients treated topically twice daily for 10 days with placebo vs. terpene mixture in DMSO. * p < 0.01 for terpene treatment at Day 10 vs. Day 0 and vs. placebo Day 10. One-Way ANOVA, Post-hoc Tukey p < 0.01.

10. It is not known if the score differences would have reached significance after day 3 but before Day 10. However, a trend is suggested, and it can be inferred that a therapeutic response initiated early after use. This trend is illustrated in Figure 3.

Parsing out data by gender, the mean pain score for men on Day 10 of active treatment was 9.4 +/− 4.6 and for women was 8.3 +/− 5.8. Using an unpaired t- test (www.graphpad.com) there was no significant difference between these means on Day 10 of active treatment. (p=0.8).

Table 4 summarizes the data. Twenty-five out of 32 (78.1%) patients had significant decreases in pain scores after 10 days. None of the placebo treatment group participants reported significant improvement in their pain scores. No adverse events were reported in either group.

**Table 4:** Pain Scores of Study Participants on Day 0, Day 1, Day 3, and Day 10.

Placebo	Active	
Pain Score – Day 0	65.4 ± 13.6^	61.1 ± 15.6^
Pain Score—Day 1	48.3 ± 16.8^	36.0 ± 12.4^
Pain Score—Day 3	51.9 ± 14.8^	26.8 ± 9.6^
Pain Score – Day 10	60.8 ± 11.2^	8.8 ± 7.2*
Gender Pain Scores – Day 10	Male (13)	9.4 ± 4.6^#^
	Female (19)	8.3 ± 5.8^#^

**^**p > 0.1 No Difference compared to Day 0.

*p < 0.01 ANOVA, p < 0.01.

post-hoc Tukey.

^#^p > 0.8 No Difference in group means. Unpaired t-test.

Questionnaire Item scores were summed, divided by the largest possible sum of the item scores which was 20, then multiplied by 100 to normalize the scores to a.

percentage of the maximum possible pain score.

## Discussion

Plantar fasciitis is the most common cause of heel pain in adults with a lifetime incidence of 10% and over 2 million adults affected annually in the US. PF has been associated with heel pain, falls, poor quality of life, and disability^3^. It is also associated with various sports, being reported in recreational and elite runners with an incidence of 5–10%^15^.

A variety of treatment options are available including physical therapy with stretching and strengthening, foot orthoses, heel padding, ice massage, NSAIDS, decreased weight bearing activity, taping, dry needling/acupuncture, night splints, extracorporeal shock wave therapy, steroid injections, and surgery. These approaches have been reviewed^1,4^. However, most treatment modalities are not supported by sufficient evidence to recommend one strategy over another^1,10^. The majority (over 75%) of patients with PF will improve with conservative non- operative treatment within 12 months^9^, but during the interim patients often suffer from significant impairment of recreational and activities of daily living^3^, as well as lost time from work^11^. Thus a rapid acting, non-invasive, and convenient modality would add greatly to current treatment options.

The use of topical analgesic agents for soft tissue pain is not new. For example, a recent study by Guo *et. al.*^16^ used a topical mix of methyl salicylate, menthol, camphor, and thymol in 3600 subjects with soft tissue pain. After 7 days of use over 70% of subjects had a large decrease in their VAS pain scores. Also by way of example, diclofenac has recently been FDA approved for topical use for osteoarthritis in a gel containing 45% DMSO^17^. The current study adapts this topical approach to soft tissue pain control, utilizing skin permeation enhancement to negotiate the unique challenges plantar fasciitis treatment presents, i.e., thick skin, fibrotic tissue, and thickened fat pad on the sole.

The average length of pain duration at study enrollment was over 4 months in both groups, indicating the majority of patients were in a chronic stage of PF, rather than acute. Patients from the physical therapy office were enrolled on the first visit and prior to beginning a supervised physical therapy regimen. The majority of patients in both cohorts were overweight or obese, and had used an NSAID, shoe inserts, or home massage/stretching within the past 30 days (Table 3). A breakdown of results by pain duration was not performed. There were too few patients in an acute phase (less than 3 weeks). The effects seen in the study thus are primarily for patients with chronic pain who have failed other non-invasive treatments.

There was no apparent gender difference in treatment response by Day 10 and we conclude this treatment works equally well in men and women.

Given the complexity of the current formulation it is not possible to determine a precise mechanism of action, nor if efficacy is due solely to some of the known active agents as discussed below or to a complex synergy or additive effect with other oil components. The anti-inflammatory and anti-nociceptive actions of a variety of essential oils has been investigated^18–25^, and purported mechanisms of action include inhibition of arachidonic acid metabolism, cytokine production, and pro-inflammatory gene expression ^12^. Other actions of the current formulation may include direct effects on pain mediation. As examples: camphor and eucalyptol (1,8-cineole) are known to desensitize the pain-mediating transient receptor potential channel type A1 (TRPA1) with resultant analgesic and anti-inflammatory effects^25–27^; the vanilloid transient receptor potential channel (TRPV) plays a central role in pain transduction^28^ and is strongly inhibited by vanillin; menthol from wintergreen oil is known to be a topical analgesic^29^ and recently has been shown to inhibit the production of pro-inflammatory cytokines such as tumor necrosis factor-alpha (TNF-α) and interleukin-6 (IL-6)^30^; eugenol (a small phenolic compound and not strictly a terpene) has a long history of use in dentistry for topical pain relief^31.32^. Tea tree oil also has reported topical analgesic properties^33^, although no effect was seen by itself in this study. These agents and their known actions may thus be responsible for the effects seen in the current study.

Advantages of the current approach include: use of agents with GRAS (Generally Recognized As Safe) status by the FDA^34^, an adaptive approach to a difficult-to-treat body area, low cost, ease of application, convenience of safe at-home use, avoidance of side effects inherent in systemic treatments, avoidance of surgery with risk of infection and prolonged recovery times, ability to achieve a high concentration of actives at the affected site while minimizing system absorption, and avoidance of steroids or opiates. Minimizing systemic absorption can greatly decrease untoward effects. Chronic use of oral NSAIDS, for example, can result in decreased renal function, GI bleeding, and poor patient compliance due to GI complaints^35^. A topical approach avoids these kinds of systemic issues.

Patient satisfaction with the protocol was high. Two patients in each group dropped out of the study, all reporting intolerance of the aromatic aroma. No patient reported an adverse event from application of either the treatment or placebo formula.

It is likely that skin permeation enhancement by limonene^36,37^, rosemary oil^38,39^, and DMSO^40^ contributed to the efficacy seen in this study. The clinical use of pharmaceutical- grade DMSO as a penetration enhancer is supported by the robust data that have accumulated over the past 3 decades demonstrating the favorable safety and tolerability profile as well as its anti-inflammatory actions^17,40^. The concentration in the current formulation of 15% delivers approximately 300ul of DMSO per day when 1ml of the formula is applied twice daily, a very modest amount.

## Limitations

Limitations of this study include the lack of racial diversity (87% white), relatively small sample size, lack of patient stratification based on activity level or amount of NSAID use, or breakdown of response by BMI. Neither plantar fat pad nor plantar fascia thickness were measured, and both of these are known to be altered in plantar fasciitis. Either or both these factors could affect the ability of the formula to reach the affected fascia. No breakdown of effect by length of pain duration, i.e., acute vs chronic, was performed. The placebo and active formulations did not have identical aromas, however it is unlikely that this had any impact on the blinding of the study protocol. The fact that each formulation contained essential oils and had an aroma gave no information to any participant about which treatment group they were allocated to.

## Conclusion

In this study we evaluated the efficacy of a solution containing essential oils with concentrations of known therapeutic agents for topical use, formulated with the skin permeation enhancers limonene and small amounts of DMSO in the treatment of chronic plantar fasciitis. After 10 days of twice daily application 78% of patients had an 85% or greater reduction in their pain scores. No adverse events were reported. An inexpensive topical approach to treating plantar fasciitis as provided in this study could greatly reduce costs to patient and society, reduce lost time from work, improve quality of life in those suffering from PF, and provide an effective first line therapy in the primary care office without need for referral, use of steroids, or use of opiates. This is a small preliminary study and further trials with larger numbers of patients of a wider diversity are required to see if results are generalizable to a broader population.

## Data Availability

The datasets used and/or analyzed during the current study are available from the corresponding author on reasonable request.
